# Characterization of hepatic lipid profiles in a mouse model with nonalcoholic steatohepatitis and subsequent fibrosis

**DOI:** 10.1038/srep12466

**Published:** 2015-08-20

**Authors:** Kosuke Saito, Takashi Uebanso, Keiko Maekawa, Masaki Ishikawa, Ryo Taguchi, Takao Nammo, Tomoko Nishimaki-Mogami, Haruhide Udagawa, Masato Fujii, Yuichiro Shibazaki, Hiroyuki Yoneyama, Kazuki Yasuda, Yoshiro Saito

**Affiliations:** 1 Division of Medical Safety Science, National Institute of Health Sciences, 1-18-1 Kamiyoga, Setagaya, Tokyo 158-8501, Japan; 2 Department of Metabolic Disorder, Diabetes Research Center, Research Institute, National Center for Global Health and Medicine, 1-21-1 Toyama, Shinjuku, Tokyo 162-8655, Japan; 3 Division of Biochemistry, National Institute of Health Sciences, 1-18-1 Kamiyoga, Setagaya, Tokyo 158-8501, Japan; 4 Stelic Institute & Co., Inc., 1-9-15 Higashi Azabu, Minato, Tokyo 106-0044, Japan

## Abstract

Nonalcoholic steatohepatitis (NASH) is a major health problem since it often leads to hepatocellular carcinoma. However, the underlying mechanisms of NASH development and subsequent fibrosis have yet to be clarified. We compared comprehensive lipidomic profiles between mice with high fat diet (HFD)-induced steatosis and STAM mice with NASH and subsequent fibrosis. The STAM mouse is a model that demonstrates NASH progression resembling the disease in humans: STAM mice manifest NASH at 8 weeks, which progresses to fibrosis at 12 weeks, and finally develop hepatocellular carcinoma. Overall, 250 lipid molecules were detected in the liver using liquid chromatography-mass spectrometry. We found that STAM mice with NASH presented a significantly higher abundance of sphingolipids and lower levels of triacylglycerols than the HFD-fed control mice. The abundance of certain fatty acids in phospholipid side chains was also significantly different between STAM and control mice, although global levels of phosphatidylcholines and phosphatidylethanolamines were comparable. Finally, increase in levels of acylcarnitines and some diacylglycerols was observed in STAM mice toward the fibrosis stage, but not in age-matched control mice. Our study provides insights into the lipid status of the steatotic, NASH, and fibrotic liver that would help elucidate the molecular pathophysiology of NASH progression.

Nonalcoholic fatty liver disease (NAFLD) is one of the major hepatic health problems in the world^1^,^2^,^3^,^4^. About 10% of NAFLD patients are reported to develop nonalcoholic steatohepatitis (NASH), in which hepatic steatosis is accompanied by inflammation and hepatocyte apoptosis^4^,^5^,^6^. NASH can lead to fibrosis, liver cirrhosis and eventually hepatocellular carcinoma. According to the “two-hit hypothesis” of NASH progression, the “first hit” is lipid accumulation in hepatocytes^6^,^7^. The “second hit” is more complicated, and is probably a combination of multiple factors including genetics, insulin resistance, oxidative stress, mitochondrial dysfunction, and inflammation.

However, the precise mechanisms of progression from steatosis to NASH have yet to be elucidated. In addition, mechanisms underlying progression from NASH to liver fibrosis and cirrhosis also remain unclear. In humans with NAFLD, free fatty acids, which undergo esterification into triglycerides, circulate at higher levels in the blood, leading to fat accumulation in the liver^8^,^9^. Another report has demonstrated that stearoyl-CoA desaturase (SCD1), an enzyme that synthesizes monounsaturated fatty acids from saturated fatty acids, plays an important role in hepatic lipid accumulation and NASH progression^10^. Moreover, depletion of the palmitate elongase, Elovl6, attenuated NASH progression in mice, and expression of Elovl6 was positively correlated with severity of steatosis and liver injury in human NASH patients^11^. However, these observations focused only on free fatty acids, and detailed differences in overall lipid profiles at different stages of the disease have not been characterized.

Lipidomics overviews a broad range of lipid metabolites, including phospholipids, sphingolipids and neutral lipids^12^,^13^. This approach is useful to identify novel biomarkers as well as to understand changes in lipid status in clinically important diseases, such as diabetes mellitus^14^,^15^. Recently, we have demonstrated the progressive change in lipid profiles in mouse and hamster models of Alzheimer’s disease and dilated cardiomyopathy^16^,^17^. We hypothesized that characterization of lipid status using lipidomics would help illuminate the mechanism of NASH progression.

The STAM mouse develops NASH, fibrosis and finally hepatocellular carcinoma^18^. This model demonstrates the pathological progression that is very similar to the human disease, in particular the rapid and step-wise progression from steatosis to NASH to fibrosis. STAM mice also suffer from very high incidence of tumour development, at a rate of nearly 100% in males. Hence, the STAM mouse is the best available model of human NASH. In the present study, we used this model to examine changes in the lipid status in NASH. Because our primary focus is the mechanism of NASH progression from simple steatosis, we compared the lipid status of STAM mice with those of HFD-fed mice that only developed ‘benign’ steatosis. Overall, we detected 250 lipid molecules from liver tissue, including 103 phospholipids, 16 sphingolipids, 112 neutral lipids, 11 free fatty acids and 8 acylcarnitines (Cars). These metabolites were categorized into seven, three, and four classes within phospholipids, sphingolipids, and neutral lipids, respectively. In addition, we also investigated temporal lipid changes in STAM mice between 8 (NASH stage) and 12 weeks (fibrosis stage) of age. Our results show the differences in the lipid profiles between steatosis and NASH and the changes in lipid status during disease progression from NASH to fibrosis. This would help clarify the underlying molecular mechanisms.

## Results

### Biological and histological characteristics of STAM mice

STAM mice exhibited hyperglycemia and hepatomegaly as observed in previous reports (Table 1)^18^. Histological examination revealed hepatocellular ballooning and steatosis at 8 weeks (Supplemental Fig. 1a-b), the age we defined to be the NASH stage. Subsequently, lobular inflammation and fibrosis in the liver were observed at 12 weeks (Supplemental Fig. 1a–c), at which we deemed the mice to be in fibrosis stage. In contrast, HFD mice did not exhibit hepatocellular ballooning or lobular inflammation (Supplemental Fig. 1a). NAFLD activity scores of both 8- and 12-week old STAM mice were significantly higher than those of age-matched HFD mice (Table 1). Our findings were consistent with those reported in our previous paper^18^ describing this NASH model in more detail, where we also reported elevated serum ALT and AST levels, and infiltration of F4/80 positive cells by immunohistochemistry.

### Lipid profile in the NASH stage

Using ultraperformance liquid chromatography-time of flight mass spectrometry (UPLC-TOFMS), we identified 250 lipid molecules overall from liver tissues (Supplemental Table 1), including 103 phospholipids, 16 sphingolipids, 112 neutral lipids, 11 free fatty acids and 8 Cars. The number of lipid molecules in each class is listed in Table 2. The relative abundance of each lipid molecules in 8- and 12-week old HFD and STAM mice are summarized in Supplemental Table 2. Levels were normalized by internal standards (see Materials and Methods).

**Figure 1:**
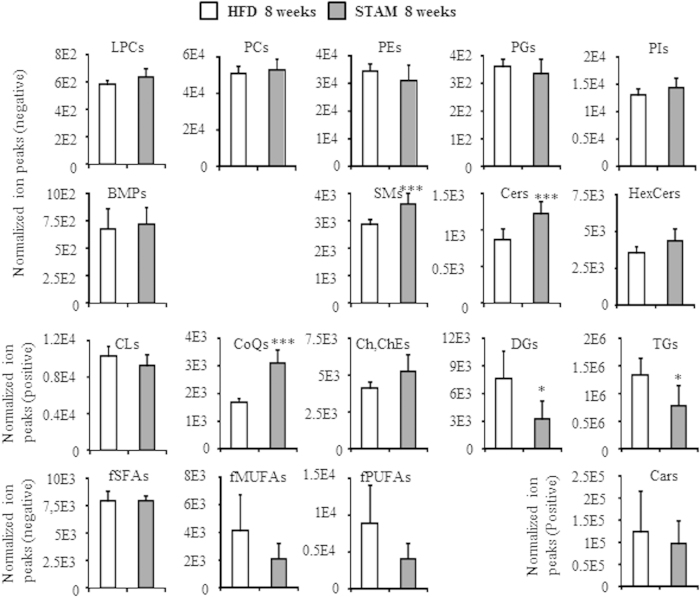
Global abundance of hepatic lipid classes in STAM and control HFD mice at 8 weeks. Lipid extracts were prepared from mouse livers as described in Materials and Methods, and abundance of lipid molecules was measured individually. Data are sum of ion peak heights of all lipid molecules within each class and shown as mean ± SD (n = 5 in each group). *p < 0.05; ***p < 0.005 in statistical tests comparing control HFD mice and STAM mice. LPCs, lysophosphatidylcholines; PCs, phosphatidylcholines; PEs, phosphatidylethanolamines; PGs, phosphatidylglycerols; PIs, phosphatidylinositols; BMPs, bis(monoacylglycerol)phosphates; SMs, sphingomyelins; Cers, ceramides; HexCers, hexosylceramides; CLs, cardiolipins; CoQs, coenzyme Qs; Ch/ChEs, cholesterol and cholesterolesters; DGs, diacylglycerols; TGs, triacylglycerols; fSFAs, free saturated fatty acids; fMUFAs, free monounsaturated fatty acids; fPUFAs, free polyunsaturated fatty acids; Cars, acylcarnitines.

To examine differences in lipid profiles between steatotic and NASH mice, we first compared the sum of the peak heights of all lipid molecules within each class. As shown in Fig. 1, no phospholipid class was significantly different in abundance between HFD and STAM mice. However, sphingomyelin (SM), ceramide (Cer) and coenzyme Q (CoQ) were significantly higher in STAM mice. In contrast, diacylglycerol (DG) and triacylglycerol (TG) were significantly lower.

**Figure 2:**
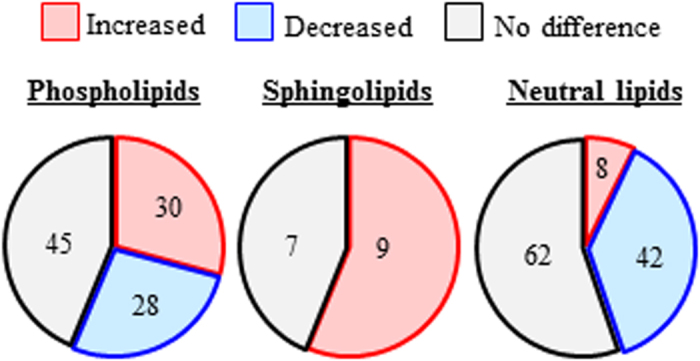
Venn diagrams of the number of lipid molecules within phospholipids, sphingolipids and neutral lipids that were significantly different in abundance between STAM and control HFD mice at 8 weeks. Increased: STAM > control, decreased; STAM < control.

On the other hand, analysis of individual molecules revealed bi-directional changes in phospholipid molecules (*i.e.*, the number of phospholipids in significantly higher levels in STAM mice was similar to the number that was significantly lower in abundance) (Fig. 2 and Supplemental Table 2). Further, sphingolipid molecules (42:1SM, 38:1Cer, 42:1Cer and 42:1HexCer) were either significantly elevated or unchanged in STAM mice, in agreement with the increased class total. Determination of fatty acid side chain revealed that 38:1Cer is C16 Cer (d18:1/16:0Cer) (Supplemental Table 2M). On the other hand, most neutral lipids such as 32:1DG and 48:0TG were found to be significantly lower in STAM mice.

These results suggest that even though phospholipid class totals are comparable, the composition of each class differed between the livers of HFD and STAM mice. To further explore this phenomenon, we determined the composition of fatty acid side chains within phosphatidylcholines (PC) and phosphatidylethanolamines (PE), the two largest phospholipid classes comprising about 30 lipids each, which are sufficient to detect differences in composition. Table 3 presents the percentage of individual phospholipids in each class, along with their fatty acid side chains. In each class, seven molecules presented a difference in abundance of more than 1% between HFD and STAM mice. It is noteworthy that these molecules contain either palmitate (16:0) or stearate (18:0) as a side chain. All phospholipid molecules containing palmitate, except 16:0/22:6PC, were less abundant in STAM mice, while those containing stearate were more so.

**Figure 3:**
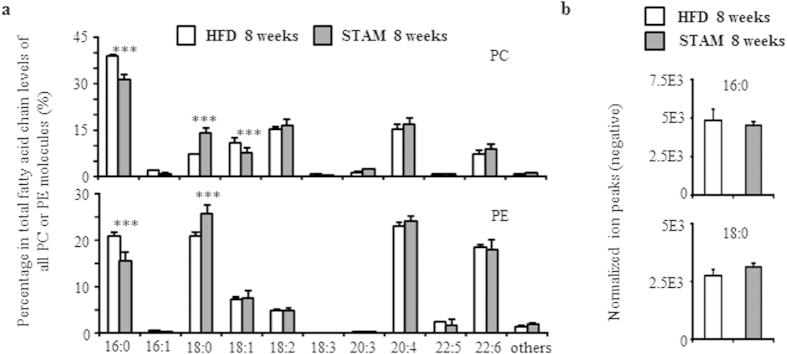
Fatty acid levels between 8-week old HFD and STAM mice. **a,** Abundance of each fatty acid as percentage of all side chains, calculated as the ratio of the sum of ion peak heights containing the fatty acid to 2× the sum of peak heights of all PC or PE (one PC or PE contains two fatty acid chains). Data are mean ± SD (n = 5 in each group). ***p < 0.005 when comparing control HFD vs. STAM mice. 16:0, palmitate; 16:1, palmitoleate; 18:0, stearate; 18:1, oleate; 18:2 linoleate; 18:3, linolenate; 20:3, eicosatrienoate; 20:4, arachidonate; 22:5, docosapentaenoate; 22:6, docosahexaenoate. **b,** Levels of free palmitate (16:0) and stearate (18:0) expressed as mean ± SD (n = 5 in each group).

For a systemic comparison, we also calculated the abundance of each fatty acid as a percentage of all side chains within PC or PE (Fig. 3a). Among PCs, palmitate and oleate (18:1) side chains were significantly lower in STAM mice, stearate side chains were significantly higher, while all other side chains were unchanged in abundance. On the other hand, palmitate and stearate side chains were significantly lower and higher, respectively, among PE in STAM mice. Notably, the abundance of free palmitate and free stearate was not statistically different between HFD and STAM mice (Fig. 3b).

Since inflammation is an important NASH symptom, we determined levels of free arachidonate (20:4) and its metabolites. Arachidonate and its lipoxygenase (LOX) metabolite, 5-hydroxyeicosatetraenoate (HETE), as well as its cytochrome P450 (CYP) metabolite, 18-HETE, were significantly diminished in STAM mice (Supplemental Fig. 2a). On the other hand, the cyclooxygenase (COX) metabolites prostaglandin D_2_ (PGD_2_), thromboxane B_2_ (TXB_2_) and 12-hydroxyheptadecatrienoate (12-HHT) were unchanged. However, when levels of metabolites were normalized to arachidonate, all COX metabolites were significantly elevated in STAM mice, but LOX and CYP metabolites were unchanged, suggesting a possible relative amplification of the COX pathway (Supplemental Fig. 2b).

### Lipid profiles in subsequent fibrosis

**Figure 4:**
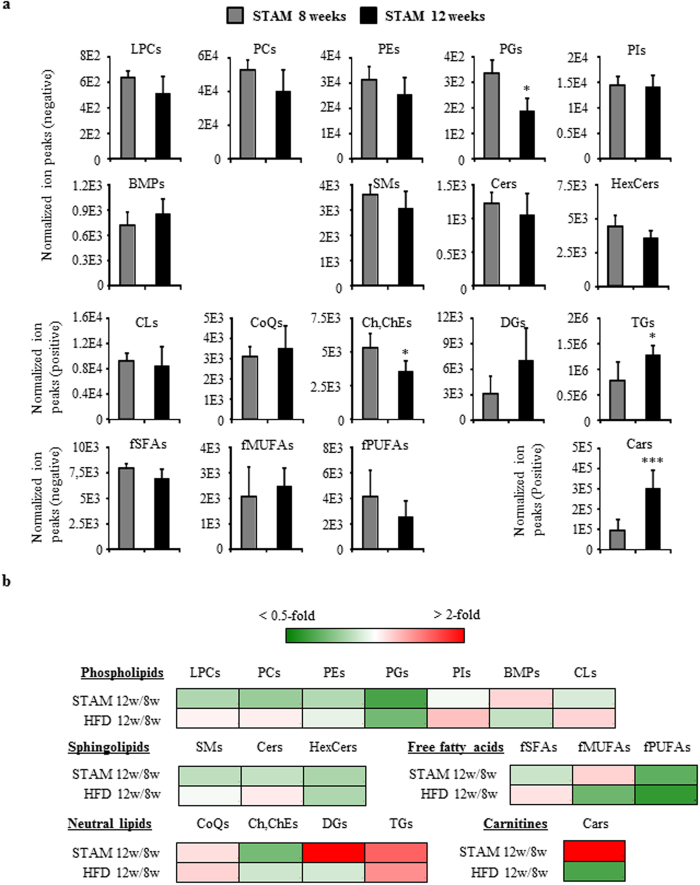
Global changes in hepatic lipid classes between STAM mice at 8 weeks and 12 weeks. **a** Lipid extracts were prepared from mouse livers as described in Materials and Methods, and abundance of individual lipid molecules were measured. Data are sum of ion peak heights of all molecules within each class, and shown as mean ± SD (n = 5 in each group). *p < 0.05; ***p < 0.005 when comparing between STAM mice at 8 weeks and 12 weeks. LPCs, lysophosphatidylcholines; PCs, phosphatidylcholines; PEs, phosphatidylethanolamines; PGs, phosphatidylglycerols; PIs, phosphatidylinositols; BMPs, bis(monoacylglycerol)phosphates; SMs, sphingomyelins; Cers, ceramides; HexCers, hexosylceramides; CLs, cardiolipins; CoQs, coenzyme Qs; Ch/ChEs, cholesterol and cholesterolesters; DGs, diacylglycerols; TGs, triacylglycerols; fSFAs, free saturated fatty acids; fMUFAs, free monounsaturated fatty acids; fPUFAs, free polyunsaturated fatty acids; Cars, acylcarnitines. **b** Heatmap showing fold change in each lipid class between age-matched control HFD and STAM mice. 8 w, mice at 8 weeks; 12 w, mice at 12 weeks.

STAM mice exhibit progression from NASH to fibrosis in a manner similar to disease development in humans^18^. Thus, to examine changes in lipid profile during disease progression, we compared STAM mice at 8 weeks (NASH stage) and 12 weeks (fibrosis stage) (Fig. 4). We also examined changes in lipid status of age-matched control HFD mice (Fig. 4b and Supplemental Fig. 3).

As shown in Fig. 4a, total Cars was significantly elevated in fibrosis-stage STAM mice, a change that was not observed in HFD mice (Supplemental Fig. 3). In contrast, a temporal increase in total phosphatidylinositol (PI) was observed in HFD mice but not in STAM mice, an observation that could be attributed to an increase in 38:4PI, the major PI molecule (Supplementary Table 2). Moreover, we observed elevated DG in STAM mice, and although this rise was not statistically significant, it was not observed in control HFD mice. As shown in Supplemental Table 2, the major DG molecules 34:1DG-a and 36:2DG significantly increased between 8 and 12 weeks in STAM mice only. On the other hand, phosphatidylglycerol (PG), cholesterol (Ch) and cholesterolester (ChE) significantly diminished, and TG significantly increased, in both HFD and STAM mice. In addition, pattern of changes in CoQ and free polyunsaturated fatty acids (fPUFAs) were similar between HFD and STAM mice, although these changes were not statistically significant in the latter.

**Figure 5:**
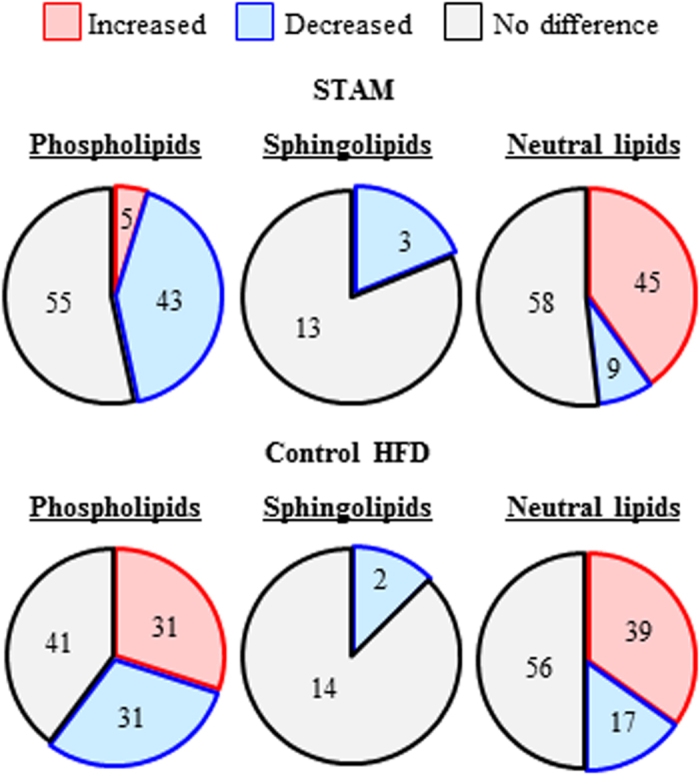
Venn diagrams of the number of phospholipids, sphingolipids and neutral lipid molecules that were statistically significantly different in abundance between STAM (upper panel) and HFD (lower panel) mice at 8 and 12 weeks. Increased: 12 weeks > 8 weeks, decreased; 12 weeks < 8 weeks.

There were 31 molecules of phophospholipids in HFD mice, and five in STAM mice, with levels that significantly increased in 12 weeks compared to 8 weeks (Fig. 5 and Supplemental Table 2). Of these, 13 molecules were PC, and two of these molecules increased in abundance in both HFD and STAM mice (17:0/20:4PC and 18:0/18:1PC, Supplemental Fig. 5). The increase in the other 11 molecules, which include 18:1/20:4PC and 18:0/22:6PC, was observed only in HFD mice.

On the other hand, seven PC molecules in HFD mice, and 12 in STAM mice, significantly decreased between 8 and 12 weeks. Of these, six molecules, including 18:2/20:4PC, 18:2/22:6PC and 20:4/20:4 PC, diminished in STAM mice only.

As for arachidonate and its metabolites, all five molecules detected were unchanged between 8 week- and 12 week-old STAM mice (Supplemental Fig. 4a), while they diminished in age-matched HFD mice (Supplemental Fig. 4b).

## Discussion

In the present study, we compared overall lipid profiles of mice at different stages of NASH progression. We used the STAM mouse, a unique diabetic mouse model with NASH that resembles the human disease in various aspects. We analysed STAM mice at the NASH (8 weeks) and fibrosis (12 weeks) stage, and compared them with control HFD mice, which only developed simple steatosis. The notable features of NASH included (1) elevated sphingolipids; (2) decreased DGs and TGs; (3) relative decrease and increase in the levels of palmitate and stearate as phospholipid side chains, respectively; and (4) increased relative abundance of COX metabolites from arachidonate. On the other hand, we observed the following characteristics in fibrosis compared with NASH: (1) increase in Cars and major DG molecules; and (2) unchanged PI, which is increased in HFD control mice.

We demonstrated that sphingolipids, including Cers, were elevated in the liver of STAM mice exhibiting NASH, while palmitate was less abundant as acyl side chains in their phospholipids. It is possible that in NASH, palmitate might be funnelled away from the synthesis of phospholipids and toward the synthesis of sphingolipids. Whether the increase in sphingolipids plays a pivotal role in NASH is presently unclear. However, it has been reported that the sphingolipid Cers have cell signalling properties relevant to inflammation, apoptosis and insulin resisitance^19^,^20^,^21^, and may also be involved in cystic fibrosis in the lung^22^,^23^. Thus, it may be reasonable to speculate that increased Cers in the NASH liver could contribute to inflammation and trigger pathological fibrosis.

Esterification of fatty acids into TG is currently thought to quench excess free fatty acids, and to prevent lipotoxicity^24^. In our NASH model, levels of TG and DG decreased in the liver. Relative insulin deficiency compared to hyperglycemic conditions was observed in STAM mice which had been treated with streptozotocin, and this may have suppressed DG and TG synthesis through down-regulation of the sterol regulatory element binding transcription factor 1 (SREBP1), because SREBP1 responds to insulin and activates DG and TG synthesis through lipogenesis and esterification of fatty acids into glycerol^25^. In fact, a report using a different model demonstrated that SREBP1 was also down-regulated in diet-induced NASH^26^, supporting the idea that suppression of TG synthesis might be involved in NASH progression.

On the other hand, phospholipids are important not only as components of the plasma membrane, but also as cell signalling messengers. For example, 16:0/18:1 PC activates peroxisome proliferator-activated receptor α (PPARα) in the liver^27^, while 18:0/18:1 PC activates it in the muscle^28^. These observations suggest that the nature of acyl side chains modulates cell signalling. In the present study, we found that palmitate-containing PC, such as 16:0/18:1 PC, decreased in abundance in NASH, but those containing stearate, such as 18:0/18:2 PC, increased. Thus, these changes might regulate hepatic function through altered cell signalling, possibly via PPARα. For example, decreased 16:0/18:1 PC may lead to insufficient PPARα activity in NASH and impair β-oxidation, which is enhanced through induction of PPARα-responsive genes^29^. This might also explain, at least in part, the accumulation of Cars in subsequent fibrosis.

In the present study, arachidonate was depleted in STAM mice at 8 weeks. Alternatively, our results further suggest that relative production of COX metabolites, but not LOX and P450 metabolites, increased in NASH. COX2 metabolites, such as PGD_2_ and TXB_2_, modulate liver injury and inflammation^30^,^31^. For example, PGD_2_ exacerbated dicloxacillin-induced liver injury by enhancing IL-4 production, and anti-TXB_2_ antibodies protect against acetaminophen-induced liver injury. It has also been reported that hepatic COX2 was elevated in mouse steatohepatitis^32^. In addition, COX2 is up-regulated in patients with cirrhosis and hepatocellular carcinoma^33^,^34^. Thus, elevated COX2 activity might be a key feature of the NASH liver. Indeed, depleted arachidonate levels in NASH may be partly due to its consumption during synthesis of PGD_2_, TXB_2,_ and 12-HHT. Another possibility is that signaling molecules other than arachidonate are mainly involved in inflammatory response in our NASH model.

Major molecules of DG, 34:1DG-a and 36:2DG, increased during the progression of NASH to fibrosis, although the change in total DG did not reach statistically significant levels. In response to various cell signals, DG is synthesized by phospholipase C from phosphatidylinositol (4,5)-bisphosphate, and the molecule acts as a secondary messenger to activate protein kinase C (PKC)^35^. Indeed, it has been reported that PKC activation by DG contributes to the development of hepatic cirrhosis^36^,^37^. Notably, PI, which is a precursor to phosphatidylinositol (4,5)-bisphosphate, increased between 8 and 12 weeks in control HFD mice, but not in STAM mice. Hence, it is possible that DG synthesis from PI is enhanced during progression from NASH to fibrosis, and thus an increase in DG may be a key step in this progression.

Our analysis also detected changes in polyunsaturated PC in STAM mice that were distinct from changes in HFD control mice over the same time frame (from NASH to fibrosis). It has been reported that supplementation with soybean polyunsaturated PC such as 18:2/18:2 PC attenuate hepatic fibrosis^38^. Although the mechanism by which the altered PC levels could induce fibrosis remains to be investigated, polyunsaturated PC could be a novel target for intervention to prevent the fibrosis that follows NASH.

Our present study showed the notable features of hepatic lipidomic status in NASH and fibrosis using STAM mice as a model, in which pathological progression is very similar to that in humans. However, there are still several concerns as to whether the lipidomic status in our model ideally mimics that in human NASH. For example, we used chemical intervention by streptozotocin in the neonatal period of the mice to develop the model. It may be also pointed out that HFD32 contains about 60 kcal% fat, which would be considered very high for humans. However, neonatal streptozotocin treatment is commonly used for generating a model of type 2, but not type 1, diabetes mellitus, and indeed our STAM mice did not show ‘absolute’ insulin deficiency, but ‘relative’ insulin deficiency compared with hyperglycemia. In addition, C16 Cer accumulation in STAM mice may reflect insulin resistance^19^. Therefore, we believe that our model shared common features with human NASH, which is often accompanied by type 2 diabetes mellitus characterized by insulin resistance and relative insulin deficiency. Thus, the lipidomic status in our NASH model, at least in part, would also be relevant for human NASH, although further studies using human subjects will be required.

## Conclusion

In conclusion, our comprehensive lipidomics approach in a mouse model of NASH revealed, for the first time, changes in lipid profile between steatosis and NASH, and between NASH and subsequent fibrosis. Understanding the molecular basis of these changes would be useful to develop novel drugs to prevent or treat NASH and fibrosis.

## Materials and Methods

### Animals

The NASH model, a STAM mouse, was generated as previously described^18^. Briefly, C57BL/6J mice were purchased from Charles River (Kanagawa, Japan) at 15 days post pregnancy. On the second day after birth, male mice were subjected to a single subcutaneous injection of 200 μg streptozotocin (Sigma, MO, USA). Four weeks after injection, mice were fed high fat diet (HFD32, CLEA JAPAN, Tokyo, Japan) *ad libitum* until sacrifice at 8 or 12 weeks. Male mice fed HFD32 without an initial streptozotocin injection were used as control HFD animals. Blood glucose and serum insulin levels were measured by a blood glucose meter (Glutest Ace, Sanwa Chemical, Nagoya, Japan) and Morinaga Ultra Sensitive Mouse/Rat Insulin ELISA Kit (Morinaga Institute of Biological Science, Yokohama, Japan), respectively. Animal experiments were conducted according to protocols approved by the Animal Research Committee at Research Institute, National Center for Global Health and Medicine. Mice were maintained according to National Institutes of Health guidelines for care and use of laboratory animals.

### Histology

Liver samples were fixed in 4% buffered paraformaldehyde, embedded in paraffin, stained with hematoxylin and eosin or Masson’s trichrome, and examined by light microscopy as previously described^18^. NAFLD activity score was calculated according to Kleiner *et al.* (2005)^39^. Results were determined as means of three different fields in each section.

### Analysis of global lipid metabolites

Lipid extraction from 10 mg liver tissue was performed as described previously^16^. Lipid extracts were loaded into UPLC-TOFMS (Waters, Milford, MA) to measure phospholipids, sphingolipids and neutral lipids as described previously^16^, and free fatty acids and Cars as follows. Free fatty acids and Cars from 5 μL lipid extracts were separated on an ACQUITY UPLC HSS T3 (2.1 × 100 mm, 1.8 μm) column (Waters). Solvent A and B were acetonitrile-water (2:3) and acetonitrile-isopropanol (1:9), respectively, with 0.1% formic acid and 0.1% of 28% ammonium hydroxide. The initial mobile phase was 27% solvent B at a flow rate of 70 μl min^−1^. A linear gradient to solvent B was run initially to 60% over 10 min, then to 80% over the next 10 min, then to 90% over another 10 min, and finally 100% solvent B over 10 min. The column was held in 100% solvent B for 15 min before re-equilibration to the initial mobile phase. The MS was operated in V-optic mode with setting scan range at 100–600 m/z from 5 to 25 min, and two functions, negative and positive ion modes, were simultaneously recorded. The MS parameters for free fatty acids and Cars were consistent with those for phospholipids, sphingolipids and neutral lipids.

UPLC-TOFMS raw data were processed using 2DICAL software (Mitsui Knowledge Industry, Tokyo, Japan), which allows detection and alignment of ion peaks of every biomolecule obtained at specific m/z and column retention time. The main parameters in 2DICAL were set as described previously^16^ with mass range from 250 to 500 m/z. Identification of ion peaks was performed as described previously^16^. Intensities of ion peaks (heights) were normalized to the following internal standards: 16:0/16:0 PC-d6 (Loradan, Malmo, Sweden) for phospholipids and sphingolipids in negative ion mode; 12:0/12:0 PE (Avanti Polar Lipid, Alabaster, AL) for Cars in positive ion mode; 16:0 lysophosphatidylcholine-d3 (Loradan) for free fatty acids in negative ion mode; and 8:0/8:0/18:2 TG (Loradan) for cardiolipin and neutral lipids in positive ion mode. Standards were added to liver homogenate prior to lipid extraction.

### Determination of fatty acid side chains in phospholipids and ceramides

Determination of fatty acid side chains in phospholipids was performed by LC-Fourier Transform Mass Spectrometry (LC-FTMS, LTQ Orbitrap XL, Thermo Fisher Scientific, Waltham, MA) as described previously^40^.

### Analysis of arachidonate metabolites

Extraction of arachidonate metabolites was performed as described previously^16^. Subsequently, metabolites were subjected to targeted analysis as described previously^17^ using a UPLC-5500QTRAP triple quadrupole-linear ion trap hybrid mass spectrometer (AB SCIEX, Framingham, MA). Intensities of ion peaks (areas) were normalized to internal standards (Leukotriene B_4_-d4, Cayman Chemical, Ann Arbor, MI), which were added to liver homogenate before metabolite extraction.

### Statistics

Body weight, liver weight, fasting blood glucose, and NAFLD activity scores are expressed as mean ± SE. To compare metabolite levels, statistical analyses of lipidomics data were performed using the Mann-Whitney U-test. Analyses were carried out using R statistical environment software (http://r-project.org/). In our analyses, *p* < 0.05 represents statistical significance.

## Additional Information

**How to cite this article**: Saito, K. *et al.* Characterization of hepatic lipid profiles in a mouse model with nonalcoholic steatohepatitis and subsequent fibrosis. *Sci. Rep.* **5**, 12466; doi: 10.1038/srep12466 (2015).

## Supplementary Material

Supplemental figures

Supplemental tables

## Figures and Tables

**Table 1:** Physiological and histological characteristics of STAM mice.

	8 weeks		12 weeks	
	HFD	STAM	HFD	STAM
Body weight (g)	25.4 ± 0.8^a^	16.7 ± 0.9^b^	34.9 ± 1.4^c^	19.2 ± 0.7^d^
Fasting blood glucose (mg/dl)	53.8 ± 2.8^a^	354.7 ± 53.3^b^	104.4 ± 9.1^c^	501.4 ± 44.7^d^
Fasting serum insulin (ng/ml)	0.222 ± 0.242	0.160 ± 0.004	0.484 ± 0.120	0.226 ± 0.041
Relative liver weight (g/kg BW)	38.5 ± 0.8^a^	56.9 ± 1.6^b^	37.2 ± 2.0^a^	73.0 ± 5.1^c^
NAFLD activity score	0.0 ± 0^a^	2.5 ± 0.3^b^	2.2 ± 0.7^b^	5.0 ± 0.4^c^

Data represent mean ± S.E. (n = 5 or 6). Significant differences were observed among groups marked with different letters, *p* < 0.05.

**Table 2:** Lipid classes detected in the liver, and number of individual lipid species within each class.

Lipid class	Ion mode	Lipid classes	Number of species
Phospholipid	Negative	lysophosphatidylcholine (LPC)	3
		phosphatidylcholine (PC)	33
		phosphatidylethanolamine (PE)	29
		phosphatidylglycerol (PG)	3
		phosphatidylinositol (PI)	9
		bis(monoacylglycerol)phosphate (BMP)	9
	Positive	cardiolipin (CL)	17
Sphingolipid	Negative	sphingomyelin (SM)	7
		ceramide (Cer)	5
		hexosylceramide (HexCer)	4
Neutral lipid	Positive	coenzyme Q (CoQ)	3
		cholesterol/cholesterolester (Ch/ChE)	6
		diacylglycerol (DG)	14
		triacylglycerol (TG)	89
Free fatty acid	Negative	free saturated fatty acid (fSFA)	3
		free monounsaturated fatty acid (fMUFA)	2
		free polyunsaturated fatty acid (fPUFA)	6
Acylcarnitine	Positive	acylcarnitine (Car)	8
		total	250

**Table 3:** The acyl side chains and abundance of individual phospholipids in PC or PE from HFD and STAM mice at 8 weeks.

PC	Side chains	% of all PC		%change	PE	Side chains	% of all PE		%change
		HFD	STAM				HFD	STAM	
32:0PC	16:0/16:0	1.06	1.09	+0.03	34:1PE	16:0/18:1	0.74	0.34	−0.40
32:1PC	16:0/16:1	1.02	0.24	−0.78	34:2PE	**16:0**/18:2	4.15	2.25	−**1.90**
32:2PC	14:0/18:2 16:1/16:1	0.17	0.13	−0.04	36:1PE	18:0/18:1	0.24	0.26	+0.03
34:0PC	16:0/18:0	0.14	0.24	+0.09	36:2PE	**18:0**/18:2	2.44	3.81	**+1.37**
34:1PC	**16:0**/18:1	16.64	8.71	−**7.93**	36:3PE	18:1/18:2	2.16	2.00	−0.17
34:2PC	**16:0**/18:2	23.37	19.91	−**3.46**	36:4PE-a	**16:0**/20:4	8.22	5.52	−**2.70**
34:3PC-a	16:1/18:2	1.40	1.01	−0.39	36:4PE-b	18:2/18:2	0.15	0.31	+0.16
34:3PC-b	16:0/18:3	1.11	0.57	−0.54	36:5PE	16:1/20:4	0.48	0.13	−0.36
35:2PC	17:0/18:2	0.18	0.35	+0.17	37:4PE	17:0/20:4	0.31	0.44	+0.13
36:1PC	18:0/18:1	1.05	1.37	+0.32	38:3PE	18:0/20:3	0.30	0.70	+0.40
36:2PC	**18:0**/18:2	3.97	7.56	**+3.59**	38:4PE	**18:0**/20:4	30.82	35.41	**+4.59**
36:3PC	16:0/20:3	2.21	3.09	+0.89	38:5PE-a	18:1/20:4	5.72	5.71	−0.02
36:4PC-a	**16:0**/20:4	19.80	15.38	−**4.42**	38:5PE-b	**16:0**/22:5	2.41	1.35	−**1.06**
36:4PC-b	18:2/18:2	0.50	1.27	+0.77	38:6PE-a	**16:0**/22:6	25.48	21.28	−**4.19**
36:5PC	16:1/20:4	0.57	0.20	−0.36	38:6PE-b	18:2/20:4	0.22	0.38	+0.17
37:4PC	17:0/20:4	0.14	0.29	+0.15	38:7PE	16:1/22:6	0.55	0.16	−0.39
38:3PC-a	18:0/20:3	0.36	1.01	+0.65	39:4PE-a	19:0/20:4	0.20	0.25	+0.05
38:3PC-b	n.d.	0.14	0.19	+0.05	39:4PE-b	19:0/20:4	0.19	0.10	−0.09
38:4PC-a	**18:0**/20:4	6.07	13.54	**+7.47**	39:6PE	17:0/22:6	0.17	0.30	+0.13
38:4PC-b	18:1/20:3	0.15	0.26	+0.11	40:4PE	18:0/22:4	0.32	0.43	+0.11
38:5PC-a	18:1/20:4	2.76	3.10	+0.34	40:5PE-a	18:0/22:5	1.16	1.00	−0.16
38:5PC-b	16:0/22:5	1.27	0.69	−0.57	40:5PE-b	18:0/22:5 20:1/20:4	0.34	0.32	−0.02
38:6PC-a	**16:0**/22:6	10.50	12.07	**+1.58**	40:6PE-a	**18:0**/22:6	5.93	9.15	**+3.22**
38:6PC-b	18:2/20:4	0.49	0.74	+0.25	40:6PE-b	18:1/22:5	0.91	0.73	−0.18
38:7PC	16:1/22:6	0.33	0.20	−0.13	40:7PE	18:1/22:6	4.67	5.21	+0.54
39:6PC	n.d.	0.06	0.14	+0.08	40:8PE	18:2/22:6	0.17	0.38	+0.21
40:5PC-a	18:0/22:5	0.48	0.46	−0.02	36:5e/pPE	16:0p/20:4	0.74	0.84	+0.10
40:5PC-b	20:1/20:4	0.17	0.18	+0.01	38:5e/pPE	18:0p/20:4	0.54	0.84	+0.29
40:6PC-a	**18:0**/22:6	2.11	3.92	**+1.82**	38:6e/pPE	18:1p/20:4	0.27	0.37	+0.10
40:6PC-b	n.d.	0.22	0.15	−0.07					
40:7PC	18:1/22:6	1.22	1.35	+0.13					
40:8PC	18:2/22:6 20:4/20:4	0.21	0.41	+0.20					
38:5e/pPC	16:0e/22:5 18:1e/20:4	0.13	0.15	+0.02					

% change = percent in STAM control mice - percent in HFD mice. Numbers in bold are changes larger than 1% Lipid species of the same formula are distinguished by letters. Abbreviation of lipid classes are listed in Table 2.
