# Supplementary material for: Characterization of hepatic lipid profiles in a mouse model with nonalcoholic steatohepatitis and subsequent fibrosis
Source: Sci Rep. 2015 Aug 20;5:12466. doi: 10.1038/srep12466 (PMC4542161; doi:10.1038/srep12466)
Supplement: Supplemental figures [file srep12466-s1.pdf]

Supplemental figures

Characterization of hepatic lipid profiles in a mouse model with nonalcoholic steatohepatitis and subsequent fibrosis

Kosuke Saito[1#], Takashi Uebanso[2#], Keiko Maekawa[1*], Masaki Ishikawa[1], Ryo Taguchi[1], Takao Nammo[2], Tomoko Nishimaki-Mogami[3], Haruhide Udagawa[2], Masato Fujii[4], Yuichiro Shibazaki[4], Hiroyuki Yoneyama[4], Kazuki Yasuda[2*] & Yoshiro Saito[1]

Supplemental figure. 1

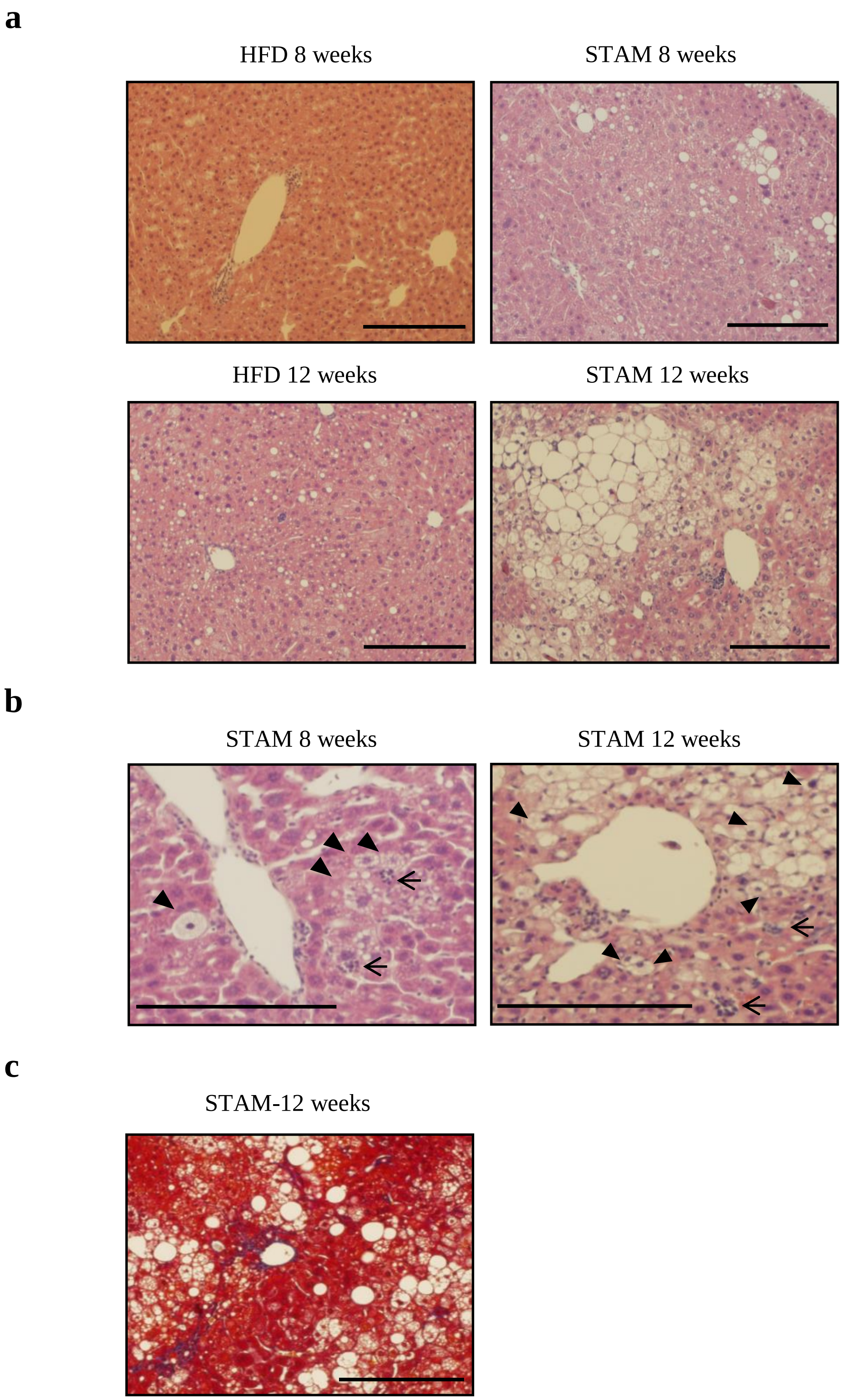

Supplemental figure 1. Histological features of the livers of STAM mice and control HFD mice. **a**, H&E stained liver sections from HFD mice and STAM mice at 8- and 12-week old. **b**, Higher power field images of liver sections from STAM mice at 8- and 12-week old. Arrow head, hepatocellular ballooning. Arrow, lobular inflammation. **c**, Masson's trichrome stained liver sections of STAM mice at 12 weeks exhibiting pericellular fibrosis. Scale bars, 200 μm.

Supplemental figure. 2

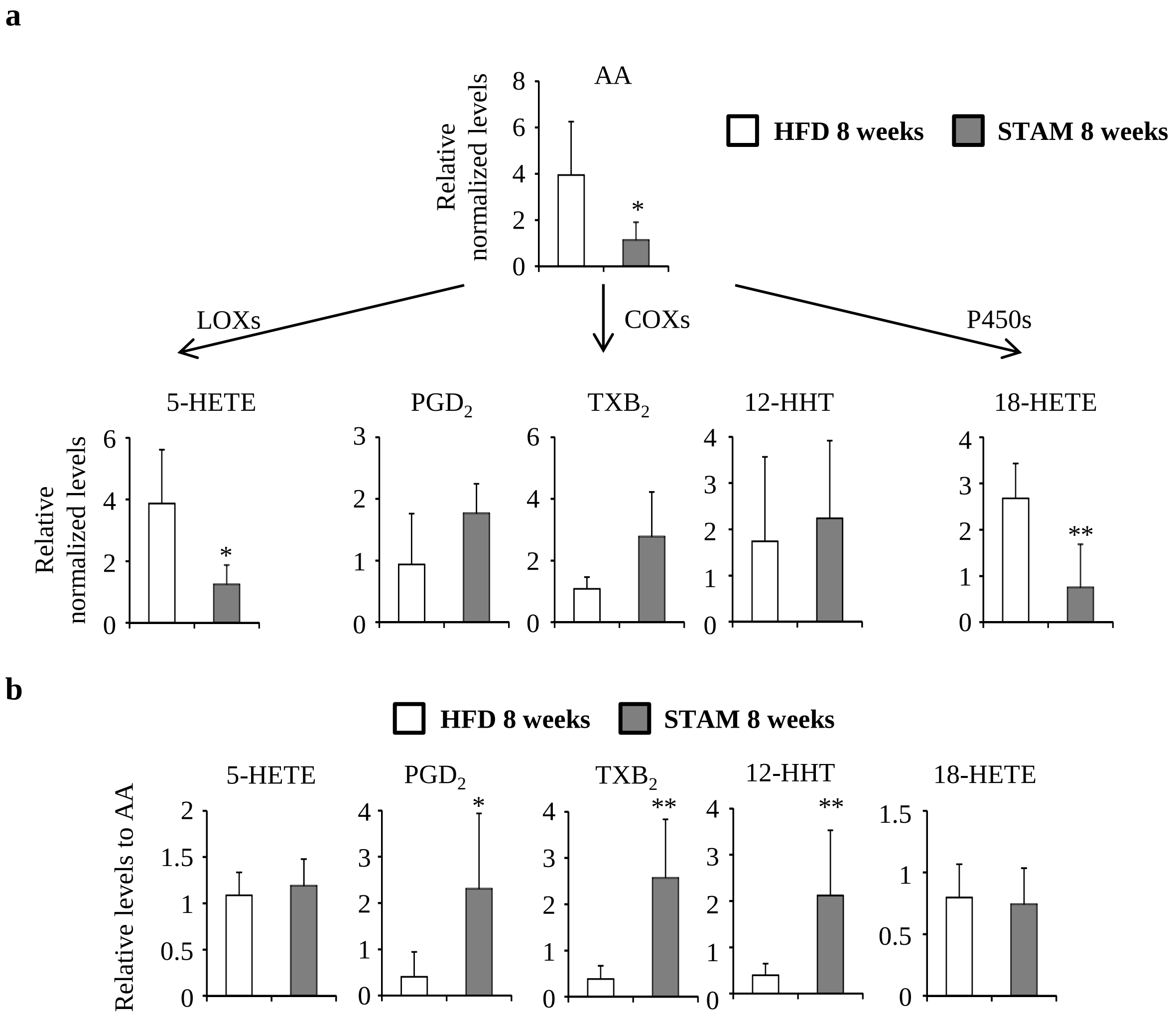

Supplemental figure 2. Differences in arachidonate metabolites between 8-week old STAM and control HFD mice. **a**, **b**. Arachidonate metabolites were measured in mouse livers as described in Materials and Methods. **a**. Data are presented as normalized level to internal standard as mean ± SD (n = 5 in each group). *, $p < 0.05$; **, $p < 0.01$ for control HFD mice vs. STAM mice. **b**. Data are presented as relative levels to arachidonate as mean ± SD (n = 5 in each group). *, $p < 0.05$; **, $p < 0.01$ for control HFD mice vs. STAM mice. AA, arachidonate; LOXs, lipoxygenases; COXs, cyclooxygenases; P450s, cytochrome P450s; HETE, hydroxyeicosatetraenoate; PGD_2, prostaglandin D_2; TXB_2, thromboxane B_2; HHT, hydroxyheptadecatrienoic acid.

Supplemental figure. 3

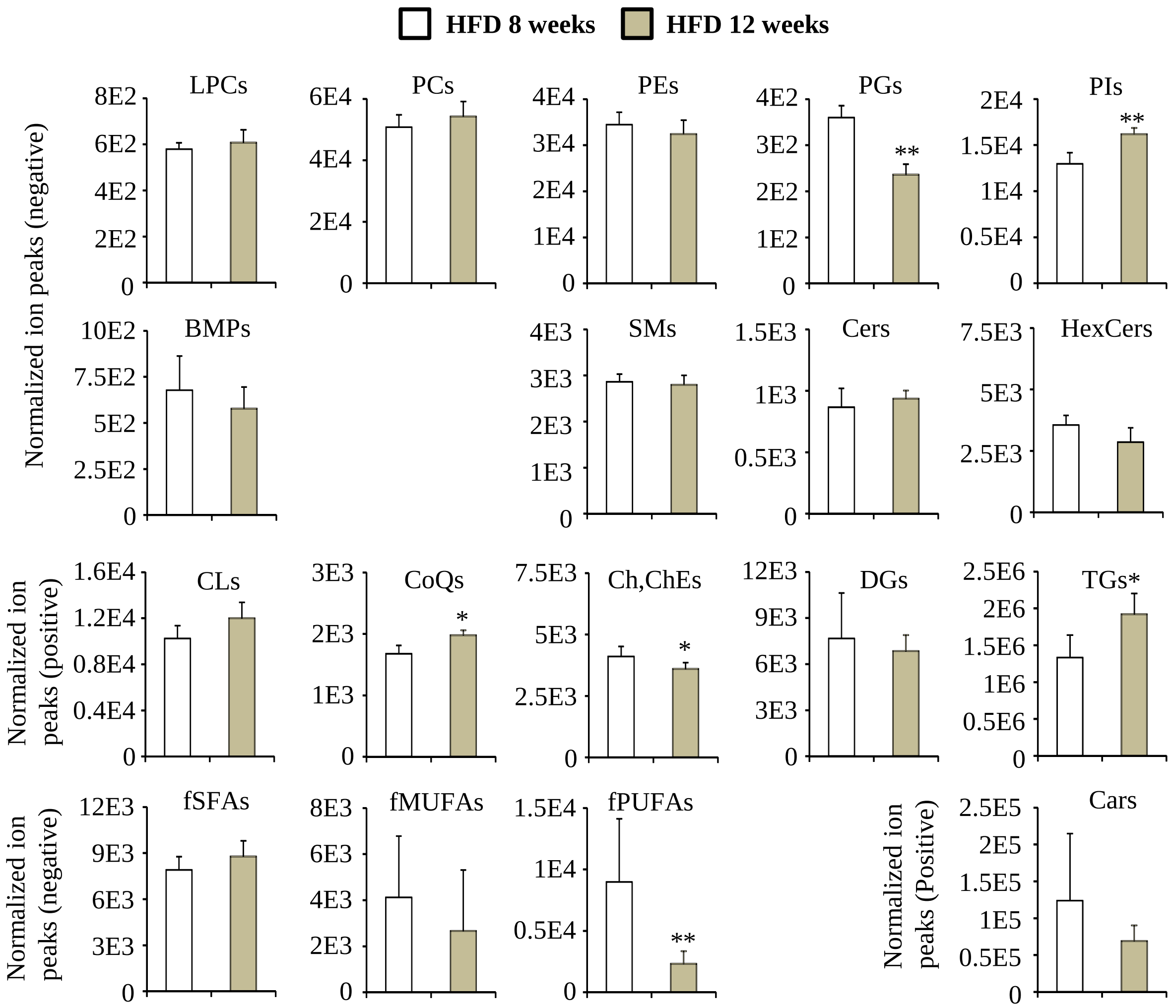

Supplemental figure 3. Global changes of hepatic lipid species between 8-week and 12-week old control HFD mice. Lipid extracts were prepared from mouse livers as descried in Materials and Methods and subjected to determination of each levels of lipid molecules. Data are sum of ion peaks of all lipid molecules within each lipid class and mean ± SD (n = 5 in each group). *, $p < 0.05$; **, $p < 0.01$ for 8-week old control HFD mice vs. 12-week old control HFD mice. LPCs, lysophosphatidylcholines; PCs, phosphatidylcholines; PEs, phosphatidylethanolamines; PGs, phosphatidylglycerols; PIs, phosphatidylinositols; BMPs, bisc(monoacylglycerol)phosphates; SMs, sphingomyelins; Cers, ceramides; HexCers, hexosylceramides; CLs, cardiolipins; CoQs, coenzyme Qs; Ch,ChEs, cholesterol and cholesterolesters; DGs, diacylglycerols; TGs, triacylglycerols; fSFAs, free saturated fatty acids; fMUFAs, free monounsaturated fatty acids; fPUFAs, free polyunsaturated fatty acids; Cars, acylcarnitines.

Supplemental figure. 4

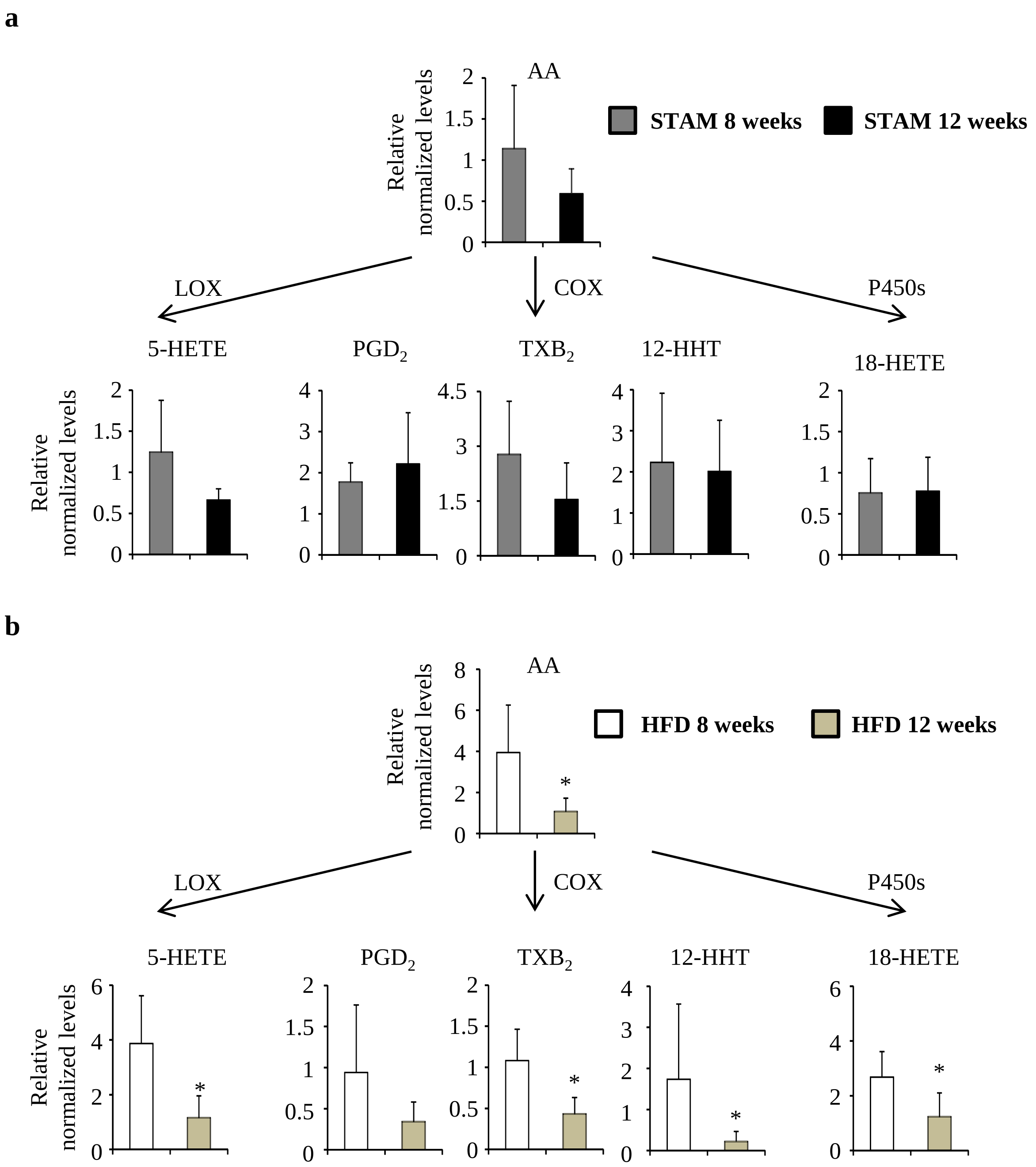

Supplemental figure 4. Differences in arachidonate metabolites between 8-week and 12-week old STAM (**a**) and control HFD (**b**) mice. Arachidonate metabolites were measured in mouse livers as described in Materials and Methods. Data are presented as normalized level to internal standard as mean ± SD (n = 5 in each group). *, $p < 0.05$ for 8-week old vs. 12-week old. AA, arachidonate; LOXs, lipoxygenases; COXs, cyclooxygenases; P450s, cytochrome P450s; HETE, hydroxyeicosatetraenoate; PGD_2, prostaglandin D2; TXB_2, thromboxane B_2; HHT, hydroxyheptadecatrienoic acid.

Supplemental figure. 5

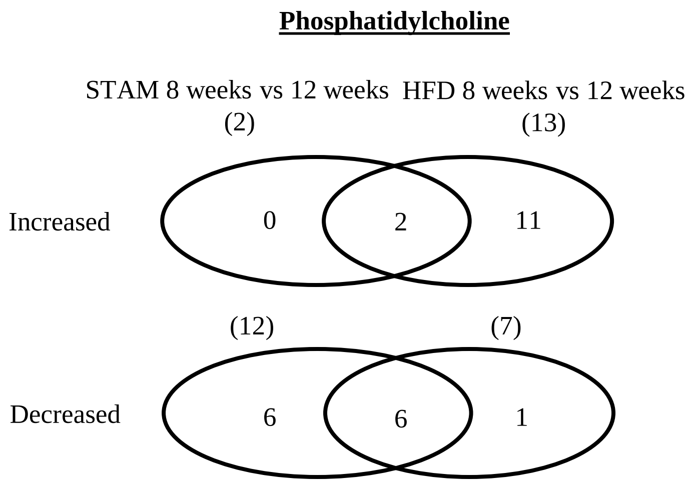

Supplemental figure 5. Venn diagrams of numbers of increased and decreased phosphatidylcholines from 8-week old to 12-week old between STAM and control HFD mice.
